# The Role of Health Technology and Informatics in a Global Public Health Emergency: Practices and Implications From the COVID-19 Pandemic

**DOI:** 10.2196/19866

**Published:** 2020-07-14

**Authors:** Jiancheng Ye

**Affiliations:** 1 Feinberg School of Medicine Northwestern University Chicago, IL United States

**Keywords:** health technology, health information system, COVID-19, artificial intelligence, telemedicine, big data, privacy

## Abstract

At present, the coronavirus disease (COVID-19) is spreading around the world. It is a critical and important task to take thorough efforts to prevent and control the pandemic. Compared with severe acute respiratory syndrome and Middle East Respiratory Syndrome, COVID-19 spreads more rapidly owing to increased globalization, a longer incubation period, and unobvious symptoms. As the coronavirus has the characteristics of strong transmission and weak lethality, and since the large-scale increase of infected people may overwhelm health care systems, efforts are needed to treat critical patients, track and manage the health status of residents, and isolate suspected patients. The application of emerging health technologies and digital practices in health care, such as artificial intelligence, telemedicine or telehealth, mobile health, big data, 5G, and the Internet of Things, have become powerful “weapons” to fight against the pandemic and provide strong support in pandemic prevention and control. Applications and evaluations of all of these technologies, practices, and health delivery services are highlighted in this study.

## Introduction

In December 2019, an emerging infectious outbreak was found in Wuhan, Hubei Province, China, and it was caused by the novel coronavirus (2019-nCoV) [1,2]. At present, the pandemic is spreading across the whole world [3]. There has been human-to-human and health care worker transmission, but the source of the coronavirus disease (COVID-19) has not been found; the route of pandemic transmission has not been fully understood. The virus may mutate, and further spread is almost certain. 2019-nCoV has a long incubation period and strong infectivity; therefore, the prevention and control of the COVID-19 pandemic faces great challenges. Compared with severe acute respiratory syndrome (SARS) and Middle East Respiratory Syndrome (MERS), COVID-19 has some new and different features; it has spread more rapidly due to increased globalization, a longer incubation period, and hidden symptoms [4].

**Figure 1:**
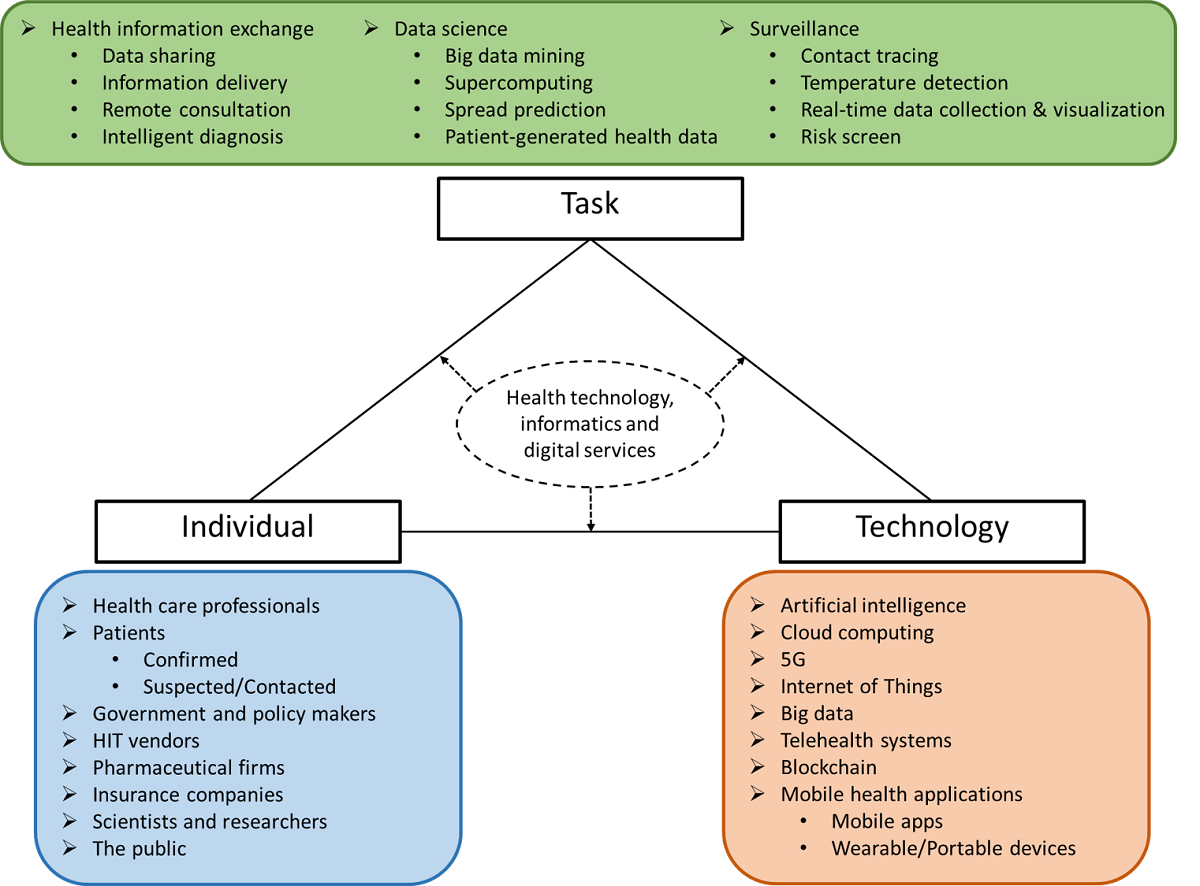
Modified fit between individuals, task, and technology (FITT) framework for health technologies, informatics, and digital services. HIT: health information technology.

Integrating novel health technologies and practices such as artificial intelligence (AI), big data, 5G mobile networks, Internet of Things (IoT), mobil health applications, telehealth services, and health information exchange (HIE) services [5] into health care systems can aid in the following ways: reporting and monitoring human transmission information, data assortment and analysis, tracking and sending alarms, etc. All of these functions are helpful to provide strong support in pandemic prevention and control. Figure 1 demonstrates a framework of health technologies, informatics, and digital services following a modified fit between individuals, task, and technology (FITT) framework [6].

## Roles and Capabilities of Health Technology and Informatics in the COVID-19 Pandemic

Taking advantage of health technologies in assisting the investigation and judgment of the pandemic will innovate diagnosis and treatment, improve service efficiency, and strengthen the capacity of information technology to support pandemic prevention and control. The specific schemes are described in the following sections.

### Health Information Acquisition and Data Analysis Application

To carry out the real-time development of pandemic tracking, key screening, and effective prediction, we should make full use of disease prevention and control information systems, and actively adopt the method of online pandemic information reporting. Big data technology will provide support for scientific prevention and policy development. The health care systems need to form a multisource platform that integrates data monitoring, exchange, convergence, and feedback mechanisms for road, railway, civil aviation, communications, medical, and other pandemic-related parties. The integrated platform will enhance the information linkage with public security, transportation, and other departments. COVID-19 diagnosis and suspected medical record collection need to be used to conduct analysis and application from the regional and national health information platform, which aids in the pandemic prevention and control, clinical treatment, and scientific research.

### Telehealth Services

With the assistance of health information technology (HIT), major hospitals, along with designated hospitals, can provide services such as remote consultation and prevention and control guidance. This will improve the ability of elementary health institutions to deal with the pandemic situation and relieve the pressure of designated hospitals.

Moreover, internet hospitals or internet diagnosis and treatment platforms have their advantages. They can provide online rediagnosis of some common and chronic diseases and drug distribution services, and reduce the risk of cross infection of patients’ offline visits. Public and standardized platforms should be adopted to gather the service links of registered and approved internet hospitals and internet diagnosis and treatment websites. This adoption would make it more convenient for people to obtain timely information on pandemic prevention and control, and diagnosis and treatment services. Organizing health care institutions at all levels to provide online compulsory counseling, home medical observation, and guidance for pneumonitis infected by COVID-19 will expand the online medical service space, guide patients to seek medical treatment, and relieve the pressure of offline clinics.

### Scientific Popularization and Real-Time Information Disclosure

COVID-19 knowledge popularization and prevention of transmission is critical to facilitate timely access to authoritative information, and assist in understanding the diseases and self-protection scientifically. Adopting national integrated online service platforms and official websites of health administration departments at all levels will be helpful to carry out pandemic information inquiries.

Social media and other popular platforms should be employed to carry out training on self-protection and for providing COVID-19 diagnosis and treatment education. This will improve the health care services and personal protection capabilities of primary care institutions.

### Health Information System Deployment

To ensure the smooth operation of the pandemic prevention and control system, an upgrade and transformation of the network should be accelerated. Where appropriate, information technology such as 5G should be applied to improve the stability and transmission quality of the designated hospitals’ network to meet the needs of patient treatment.

On the other hand, pandemic monitoring and analysis, virus tracing, patient tracking, personnel flow, and community management are important tasks during the pandemic. Health technologies such as big data, cloud computing, and AI can be applied to develop scientific strategies for precision prevention and control of the pandemic. Internet platforms can help to match the supply and demand of medical and pandemic prevention materials precisely. In this way, coordinated deployment and recycling management can be achieved. Information technology enterprises and medical research institutions should work jointly to tackle key problems, accelerate the detection and diagnosis of COVID-19 infection, and conduct research and development of new vaccines.

## Health Technology Applications and Digital Service Practices

### Artificial intelligence (AI)

As 2019-nCoV has the characteristics of strong transmission and weak lethality, the large-scale increase of infected people may drag down the medical system, so the pandemic prevention and control needs to track and manage the health status of residents and isolate suspected patients. AI’s capacity of in-depth mining and processing massive information has been used to detect and predict the spread of viruses in pandemic situations [7,8] by building intelligence monitoring platforms and developing advanced algorithms such as neural networks, the susceptible-exposed-infectious-removed (SEIR) model, and long short-term memory (LSTM) networks [9-12]. For instance, data from social media, contact tracing, surveys, etc [13] could be applied to various machine learning or deep learning models to predict the course of COVID-19 and potential reappearances [11].

After the outbreak of COVID-19, the false negative outcome of the kit test increased the difficulty of diagnosis. AI has become a powerful supplement to kit detection. The increasingly mature AI medical imaging technology [14], through tagging a large number of medical image samples and applying them to the algorithms for training, learning, and understanding, could effectively assist doctors in decision making. Furthermore, AI is also on the frontlines of the pandemic. For instance, intelligent robots are collections of integrated multi-sensor fusion, path planning, robot vision, intelligent control, and human-computer interface technology. They can provide diverse services such as disinfection, food delivery, and medicine delivery. In the situation of scarce protective clothing, the pressure on the frontline health care workers to diagnose and treat can be relieved to a certain extent, and the chance of cross-infection can be reduced.

### Telemedicine, Telehealth, and Telecommunications Technology

Telecommuting has been widely used in industries and businesses by replacing traditional commuting with digital technologies [15,16]. In the health care domain, telemedicine has been leveraging telecommunications technologies to make use of HIT and medical information such as video imaging, thus, allowing health care providers to work remotely [17,18]. Telehealth has been interchangeably used with telemedicine [19,20], but as an umbrella term, telehealth incorporates the functions of telemedicine and a variety of nonclinical services like tele-pharmacy and tele-nursing [21]. The emergence of telehealth in the coronavirus pandemic dates back to SARS in 2003 [22]. During the SARS period, to reduce people’s mobility and cross infection, a lot of work was conducted through the internet, telehealth began to show its usefulness. However, due to the network quality and technical level at that time, telehealth was limited to simple online or telephone consultation, which was relatively elementary.

Relying on the rapid development of the telecommunication technologies, video imaging, 5G, and other technologies in recent years, telehealth has developed rapidly. At present, the scenarios of remote expert consultation and remote medical education have been widely applied. Telehealth can solve the problem of unbalanced development of medical resources among regions. Through the internet, experts can organize remote consultation in remote areas and resource-deficient areas. Textbox 1 presents two basic models of telehealth.

Telehealth has brought the benefits of high-quality medical resources from superior hospitals to the community-level health institutions, and it has greatly improved health quality, efficacy, efficiency, and saved expenses.

Remote diagnosis through internet technology and big data enables patients to perform imaging and electrocardiogram examinations at township-level hospitals, and doctors at grassroots hospitals can transmit information to their superiors via the internet. The regional diagnosis center and experts will issue a timely diagnosis report, which is convenient for doctors at the elementary hospital to provide patients with targeted treatment.

During the COVID-19 pandemic, telehealth systems can assist and support health care professionals to conduct remote consultation so that doctors from different regions can converge together to discuss the diagnosis and treatment of patients [23]. The system will connect the remote mobile workstation beside the patient’s bed, and real-time video meetings can be carried out so that the experts can observe the actual situation of the patient. During the process, patients have interactions through video [24] or telephone [25] with health care providers [26]. The history of symptoms and exposure risk will be obtained through an observational assessment. Based on this information, doctors can make judgments on whether the patient has been infected or needs further testing. Telehealth has also been used to help manage the patients with suspected symptoms of COVID-19 and provide virtual medical services for chronic diseases [27,28]. The image diagnosis system digitizes the results generated by the examination equipment (x-ray, ultrasound machine, etc) in the medical institution; health care providers can access the electronic health records (EHR) remotely through the network to realize remote diagnosis. For patients who are critically ill, telehealth intensive care equipment transmits the physiological information and medical parameters to the monitoring center through the telecommunication network, and real-time detection and further analysis can be conducted. The systems can realize interoperability of automated health data through HIE [5] and the sharing of medical information with participating hospitals. Telehealth shortens the distance between doctors and patients, and helps doctors provide timely medical services based on physiological information transmitted from distant places [29]. Experts from other places remotely access the EHR of patients in the insulation ward, discuss the treatment plan and effect in real time, and give professional opinions.

Given the shortage of medical protective material and personal protective equipment (PPE), the use of remote consultation reduces the occurrence of on-site diagnoses, which also saves PPE, and reduces the risk of infection spread caused by the transfer of diagnosed patients to the superior hospitals.

**Basic models of telehealth.**

**Direct docking mode**

If the inviting medical institution finds the invitee on its own, then telehealth can be carried out directly between the medical institutions.

**Platform matching mode**

If the inviting medical institution cannot find the invitee by itself, it can publish the requirements on the remote medical service platform established by the inviting medical institution or a third-party institution. The platform can match the invitee’s or other medical institutions’ initiative to the inviter’s needs of health response.

### 5G

The fifth generation mobile networks, or fifth generation wireless systems, is the latest generation of cellular mobile communication technology. It provides at least 10 Gbit/s peak rate and millisecond-level transmission delay [30]. The network enables a new kind of network that is designed to connect everything together virtually, including human and machines, objects and devices with high reliability and capacity. Compared to prior generations, 5G has larger bandwidth, higher rate, lower delay, and larger connection. 5G communication technology can not only achieve high-quality transmission of 3D images but also provide services such as data acquisition, real-time positioning, remote diagnosis and treatment, and other fusion functions in addition to information communication [31].

In a prior generation of wireless systems such as 4G, network bandwidth was limited, only meeting the transmission of small volumes of medical information. For medical images like computed tomography scan images [23], real-time remote consultation, and telemedicine meetings, the network must have high transmission speed and low latency. The COVID-19 pandemic is a global emergency; saving time means saving patients’ lives.

In the medical industry, communication is one of the important factors that impact the development of medical rescue. The high-speed communication capacity of 5G can effectively improve the efficiency of medical emergency rescue and the response ability at public health events.

Remote diagnosis and treatment based on 5G between doctors and patients can be realized through real-time high-definition audio and video connection. Medical data transmission can promote the transition from “face-to-face” consultation to video remote consultation, which further improves efficiency and precision. Under the current situation of the pandemic, 5G and telehealth can make diagnosis and treatment more efficient, convenient, and safe. The high-speed, large-capacity, and low-latency of the 5G network accelerate achieving the needs of real-time, high efficiency, and stability of the remote consultation.

5G telecommunication technologies also improve the health care accessibility. Health information systems gather clinical data from various locations such as hospitals, community health care organizations, and physician practices. 5G supports real-time health data exchanging so that health care professionals can get access to patients’ diagnosis records, medical history, and lab results without delay and information transmission barriers. Prior generation of wireless systems could not provide such capacities for HIE [32] and may cause health care workforce burnout [33]. With the implementation of 5G, the traditional medical workflow will be improved dramatically, and the unnecessary contact between health care providers and patients may be reduced. The electronic health information system can track the whole process of the medical order, which decreases the risks of medical errors and improves the quality of health care and system management.

In the prevention and control of COVID-19, it is essential to make full use of 5G communication technology, linking doctors and experts across the country and even around the world; actively taking advantage of online diagnosis and treatment; carrying out online consultation, health science popularization, psychological assistance counseling, and home isolation guidance services such as delivery of medicines for chronic diseases; and enabling patients to receive health care without leaving home, all of which will reduce the risk of being exposed to the virus.

### Internet of Things (IoT)

IoT is a novel paradigm of interrelated digital machines, mechanicals, computing devices, and other objects [34,35]. Based on the communication protocols, it combines with the internet to realize the intelligent management of information. By taking advantage of communication technologies such as networks or the internet and sensors, everything could be linked together to realize the connection between people and objects or objects and objects. At the same time, people-oriented information, remote monitoring and control, and intelligent management are realized. Remote monitoring [36] based on IoT is an effective way to assist in achieving real-time, continuous, and long-term monitoring of patient vital signs and transfers the acquired health data or critical alarm information to health care professionals. Remote monitoring can also achieve real-time data acquisition and analysis of patients in isolated areas. Heart rate, breathing, and other physiological indicators [37] of patients with COVID-19 can be collected in the isolated zone through smart devices [38], electrocardiographs, ventilators, and sphygmomanometers; data can then be sent back in real time through Bluetooth [37] or the network. The collected data of patients’ physiological signs can be analyzed and processed intelligently in the clinical decision support system. Once the system finds abnormal data, it will send an alarm [39] in real time, and doctors will investigate and judge the situation according to the alarm information.

### Mobile Health Apps

Patient-generated health data (PGHD) are becoming more and more important in health care, especially in the COVID-19 pandemic. Coronavirus tracking apps on smartphones are playing critical roles as a mobile technology to collect, gather, and share PGHD during the pandemic. Users can check whether they have had contact with patients who are infected by entering personal information. When registering, users are required to enter their name as well as age, zip code, and other relevant information. Phone numbers are also recorded, so users will be notified once the system identifies contact with patients who are infected. Most trace tracking apps use network or Bluetooth technologies. When other users get close to a certain social distance range, the app will perform digital handshakes with them; this “interaction” will be recorded and encrypted [40]. This information is useful to identify high- and potential-risk groups, which further accelerates conducting accurate investigations, prevention, and monitoring [41]. During the pandemic, apps for tracking, tracing, and early warning have been successfully implemented in many countries to control the spread of the coronavirus. Tracking the flow of personnel helped predict the spread trend of the pandemic and improved the efficiency of prevention and control work.

However, ethical and legal issues [42] are raised with the widespread use of contact tracing and monitoring technologies [43]. The pressure to protect personal information is higher than ever. User data is a “disaster area,” as privacy can be leaked. Some apps claim that data will be encrypted on the user’s mobile phone for a few days and then permanently deleted if users have not been exposed to patients who are infected. Some apps need to obtain the user’s consent on two aspects: user’s agreement with data being collected while using the app and agreement on releasing relevant personal data once they are found to have contact with patients who are infected.

Data opening and sharing in the context of pandemic prevention and control cannot exceed the reasonable limits. The disclosure and use of data need to protect the rights and interests of citizens to achieve a dynamic balance between public governance and citizen protection. On the one hand, making good use of data technology has been proven to optimize the governance of the public health emergency; on the other hand, personal information and privacy data protection boundaries should be strictly guarded to avoid unnecessary and irreparable damage. Technical tools such as data desensitization and blockchain technologies should be applied along with detailed personal information classification standards, strict privacy regulations, and policies.

### Big Data

With the threat of the COVID-19 pandemic, an urgent public health crisis that produces massive data from multiple sources, the use of big data technology can provide the public and decision makers with more complete, continuous, accurate, and timely pandemic prevention information and traceable disease source-based methods [44].

Through big data technology along with geographic location and time stamp information, it is possible to analyze the movement trajectory of affected persons [45]; comprehensively track the movement trajectories of patients who are infected, suspected patients, and related contacts; and accurately describe the cross-regional infiltration. The health care database could be integrated with immigration and customs data to generate real-time alarms during clinic visits to assist in identifying infected cases [46]. Movement tracking has provided powerful data support for the prevention and control of the pandemic.

## Conclusion

Currently, the prevention and control of COVID-19 is in a critical period. The construction of integrated intelligent health care systems through novel health technology applications plays a vital role in blocking the spread of the pandemic. The intelligent health care system integrates strategic emerging health technologies and health care delivery services and practices, such as AI, big data, 5G, IoT, cloud computing technology, sensor technology, telehealth service, mobile health apps, and HIE practices. This system will form a new mode of innovation and upgrading of traditional medical and health informatization.

Meanwhile, it is also critical to establish security and privacy protecting systems to enhance the infrastructure, medical data, and management system. It is important to prevent patient information leakage, create a safer and more convenient medical treatment environment, and provide a strong security guarantee for the intelligent health care system.

Through combining health technology and health care systems, diagnosis efficiency and patients’ medical experiences can be improved, and remote sharing of high-quality medical resources and real-time information interaction can also be achieved. The integrated system can effectively alleviate the problems of medical resource shortages, uneven distribution of health care quality, and shortages of health care workers. The establishment of an integrated intelligent health care system for COVID-19 pandemic prevention and control will also provide a positive reference for the design and development of subsequent intelligent health care platforms for other public health crises.

## Abbreviations

AI: artificial intelligence
COVID-19: coronavirus disease
EHR: electronic health record
HIE: health information exchange
HIT: health information technology
IoT: Internet of Things
LSTM: long short-term memory
MERS: Middle East Respiratory Syndrome
PGHD: patient-generated health data
PPE: personal protective equipment
SARS: severe acute respiratory syndrome
SEIR: susceptible-exposed-infectious-removed
2019-nCoV: novel coronavirus
